# Adequacy of risk of bias assessment in surgical vs non-surgical trials in Cochrane reviews: a methodological study

**DOI:** 10.1186/s12874-020-01123-7

**Published:** 2020-09-29

**Authors:** Ognjen Barcot, Matija Boric, Svjetlana Dosenovic, Marija Cavar, Antonia Jelicic Kadic, Tina Poklepovic Pericic, Ivana Vukicevic, Ivana Vuka, Livia Puljak

**Affiliations:** 1 Department of Surgery, University Hospital Split, Split, Croatia; 2 Department of Anesthesiology and Intensive Care, University Hospital Split, Split, Croatia; 3 Department of Radiology, University Hospital Split, Split, Croatia; 4 Department of Pediatrics, University Hospital Split, Split, Croatia; 5 Cochrane Croatia, University of Split School of Medicine, Split, Croatia; 6 Postgraduate doctoral program, University of Zagreb School of Dental Medicine, Zagreb, Croatia; 7 Laboratory for Pain Research, University of Split School of Medicine, Split, Croatia; 8 Center for Evidence-Based Medicine and Health Care, Catholic University of Croatia, Ilica 242, 10000 Zagreb, Croatia

**Keywords:** Surgery, Risk of bias, Cochrane, Systematic reviews

## Abstract

**Background:**

Bias in randomized controlled trials (RCTs) can lead to underestimation or overestimation of the true effects of interventions. Surgical RCTs may suffer from the risk of bias (RoB) that is avoidable in trials of other interventions, and vice versa. We aimed to compare the adequacy of RoB assessments in surgical versus non-surgical RCTs included in Cochrane reviews and to assess the most common differences in those RoB assessments. Due to specificities of surgical trials, i.e. difficulties associated with blinding of surgical interventions, we hypothesized that assessments of surgical trials may be more adequate, compared to RCTs of non-surgical interventions.

**Methods:**

This was a methodological study, analyzing methods of published Cochrane systematic reviews. Data were extracted from RoB tables in Cochrane reviews (judgments and accompanying explanatory comment) for the following four RoB domains used in the 2011 Cochrane RoB tool: randomization, allocation concealment, blinding of participants and personnel, and blinding of outcome assessors. We defined adequate assessments as those that were in line with instructions from the Cochrane Handbook for Systematic Reviews of Interventions. The prevalence of adequate assessments was compared in surgical versus non-surgical trials. The most common differences in both groups of reviews were presented.

**Results:**

In 729 analyzed Cochrane reviews, there were 10,537 included trials. The prevalence of adequate RoB judgments made by Cochrane authors ranged from 87.9, 95%CI (87.3 to 88.6%) for randomization to 70.7, 95%CI (69.8 to 71.5%) for blinding of participants and personnel. For all analyzed RoB domains, the prevalence of adequate RoB domains was higher in surgical trials than in non-surgical trials. For two RoB domains assessing blinding, this difference between surgical and non-surgical trials was statistically significant (*P* < 0.001), while the difference was not significant for the RoB domain regarding randomization (*P* = 0.124) and allocation concealment (*P* = 0.039, β < 0.8).

**Conclusions:**

RoB judgments were more in line with instructions from the Cochrane Handbook when Cochrane reviews assessed surgical trials, compared to those that analyzed non-surgical interventions. However, further steps are warranted to scrutinize RoB assessment in trials of both surgical and non-surgical interventions.

## Background

Randomized controlled trials (RCTs) are crucial for assessing the effects of interventions, but various types of bias in RCTs can lead to underestimation or overestimation of the true effects of interventions [1, 2]. Therefore, Cochrane reviews of interventions include mandatory risk of bias (RoB) assessment of included trials. In the 2011 version of the Cochrane RoB tool, there were seven domains of RoB assessment for RCTs [3].

It has been reported that few RCTs in a certain surgical field have low RoB [4, 5]. Gurusamy et al. have reported that blinding is difficult in RCTs of surgical interventions, but that careful RCT design may reduce bias related to lack of blinding of surgeons and surgical staff. Gurusamy et al. also suggested that it is possible to conduct RCTs in the field of surgery with low RoB and that better understanding of RoB may result in better trials, with a better estimate of the true effects of interventions [6].

However, RoB assessments made by authors of published systematic reviews should not be taken at the face value, as we have shown in multiple studies that RoB assessments in many Cochrane reviews were inadequate and inconsistent [7–14]. Due to the specificities of surgical trials, we hypothesized that assessments of surgical trials may be more accurate and more consistent, compared to RCTs of non-surgical interventions.

The aim of this study was to compare the adequacy of RoB assessments in surgical versus non-surgical RCTs included in Cochrane reviews and to assess the most common inadequate judgments in those RoB assessments.

## Methods

### Study design and protocol

This was a primary methodological study, analyzing methods used in published Cochrane reviews, reported in accordance to STrengthening the Reporting of OBservational studies in Epidemiology [15] (STROBE Statement – Supplementary file 1).

### Inclusion and exclusion criteria

Cochrane reviews published between July 2015 and June 2016 in the Cochrane Database of Systematic Reviews (CDSR) were analyzed. This was a convenient one-year sample based on our previous studies [7, 8, 10, 16], from the period of 4 years after introduction of the 2011 RoB tool. The reviews that have included RCTs only, or both RCTs and non-randomized studies were found eligible. Diagnostic reviews, overviews, empty or withdrawn reviews, as well as those that included only non-randomized studies, were excluded.

### Screening for study eligibility

After exporting records from the Cochrane library, titles with or without abstracts of Cochrane reviews were assessed by the first author (OB) and verified by the third author (SD).

### Definition and categorization of interventions

Trials were categorized as surgical, conservative, or mixed depending on the invasiveness of its intervention (or comparator). For the purpose of this study, invasiveness was considered as something which requires close contact between the person administering the procedure and the person who requires the procedure. Strictly invasive (or surgical) procedures are medical procedures that invade (enter) the body, usually by cutting or puncturing the organs (primarily skin, but other organs as well as mucosa, teeth, etc.) or by inserting instruments into the body through these cuts. Apart from downright surgical procedures, this category includes comparisons of different surgical techniques, dental interventions, different ERCP (endoscopic, retrograde cholangiopancreatography), or EMS (extra-mucosal resection) techniques.

Less invasive (unclear or mixed) procedures involve entry into a body cavity or interruption of normal body functions and may include puncturing the skin, administration of non-oral medication, insertion of a tube or medical devices, and care following medical procedures, such as stoma care and catheter care. Examples are acupuncture, external manipulation with the fetus (ECV – external cephalic version), different modalities of artificial ventilation/respiration (e.g. CPAP – continuous positive airway pressure or HFJV – high-frequency jet ventilation), types of anesthesia (block, spinal, general), application of different types of catheters without the change in the application technique (unless technique clearly surgical), interventions simulating invasive techniques (virtual trainer), an invasive procedure not strictly surgical (puncture of fluid collection, intraarticular injection).

Every other intervention with no proof of invasiveness was considered conservative or non-invasive. This also includes interventions or different procedures before or after surgical treatment (different physical therapy or medications) that do not change the performance of the surgical procedure being planned or applied at that moment.

Interventions were categorized by two authors. The first categorization of interventions was performed during analysis of the domain for blinding of participants and personnel; for verification, categorization was repeated independently during the analysis of the domain for blinding of outcome assessors. Discrepancies and missing categorizations were resolved by the first author.

We made the decision to merge mixed and non-surgical categories due to the observed raw agreement of the two independent categorizations and the best inter-rater agreement. Finally, the trials were divided according to interventions to surgical or non-surgical. Details about categorizations and inter-rater agreement are presented in Supplementary Table 1.

### Data extraction

The following data from RoB tables were extracted: trial name, judgment (RoB is low, unclear, or high), and explanatory comment for each judgment. For data extraction, automatic data scraping from the Cochrane library was used, designed by the first author (OB), as described previously [7].

### Assessment of adequacy for four domains of risk of bias tool

In each eligible trial of the included reviews, an assessment of whether judgments of Cochrane authors were adequate was made for the following four RoB domains: random sequence generation, allocation concealment, blinding of participants and personnel, and blinding of outcome assessors. The adequacy of judgments was analyzed by comparing original Cochrane authors’ judgments with our reassessed judgments; instructions from the Cochrane Handbook for Systematic Reviews of Interventions [17] were considered a gold standard for making judgments. The source for our assessments was the accompanying comment from the RoB table and description of the intervention provided by the Cochrane authors. For the RoB domain for random sequence generation and allocation concealment accompanying comments were categorized to bring judgment as described in our previous studies [8, 10]. For the blinding domains, we needed to determine which subject was blinded and whether the outcome(s) were susceptible to lack of blinding [7, 16]. In the final stage, the prevalence of inadequate assessments was compared and reasons for inadequate RoB assessments between surgical and non-surgical trials stated.

### Primary outcome

The primary outcome was the prevalence of inadequate RoB judgments for four Cochrane RoB domains in surgical versus non-surgical trials.

### Secondary outcomes

The secondary outcomes were the distribution of RoB judgments (low/unclear/high) and the prevalence of various reasons for inadequate assessments in surgical versus non-surgical trials.

### Statistics

Descriptive data were presented as frequencies and percentages. Prior to analysis datasets were tested for normality by the Kolmogorov-Smirnof test. For non-parametric data, the Wilcoxon test was used for paired samples, Mann-Whitney test for comparison of two independent samples while the Kruskal-Wallis test was used for comparison of three or more samples. When the Kruskal-Wallis test was positive (*P* < 0.05) a pairwise comparison of subgroups was performed according to Conover. No adjustments of *p*-values in post hoc analyses were considered due to our study being exploratory and involving post-hoc testing of unplanned comparisons [18]. For the same reason, with the idea of emphasizing emerging hypotheses regarded for further investigation, if post-hoc analysis did not detect the differences in pairwise comparison, one-way ANOVA was reapplied on the dataset instead of the Kruskal-Wallis test and Student-Newman-Keuls test for pairwise comparison of subgroups was used. The difference in proportions was tested with the Chi-squared test. For all statistical tests we used type I error α = 0.05, and type II error β = 0.2. Statistical analyses were performed using MedCalc for Windows, version 12.5.0.0 (MedCalc Software, Ostend, Belgium). We calculated the raw agreement and presented it along with Cohen’s unweighted kappa with corresponding 95% CI (confidence interval) as a measure of inter-rater agreement [19]. We classified the level of agreement as follows: values ≤0 as indicating no agreement and 0.01–0.20 as none to slight, 0.21–0.40 as fair, 0.41–0.60 as moderate, 0.61–0.80 as substantial, and 0.81–1.00 as almost perfect agreement. Outcomes, hypotheses, statistical tests with respective results, and conclusions are presented in Supplementary tables.

## Results

**Fig. 1:**
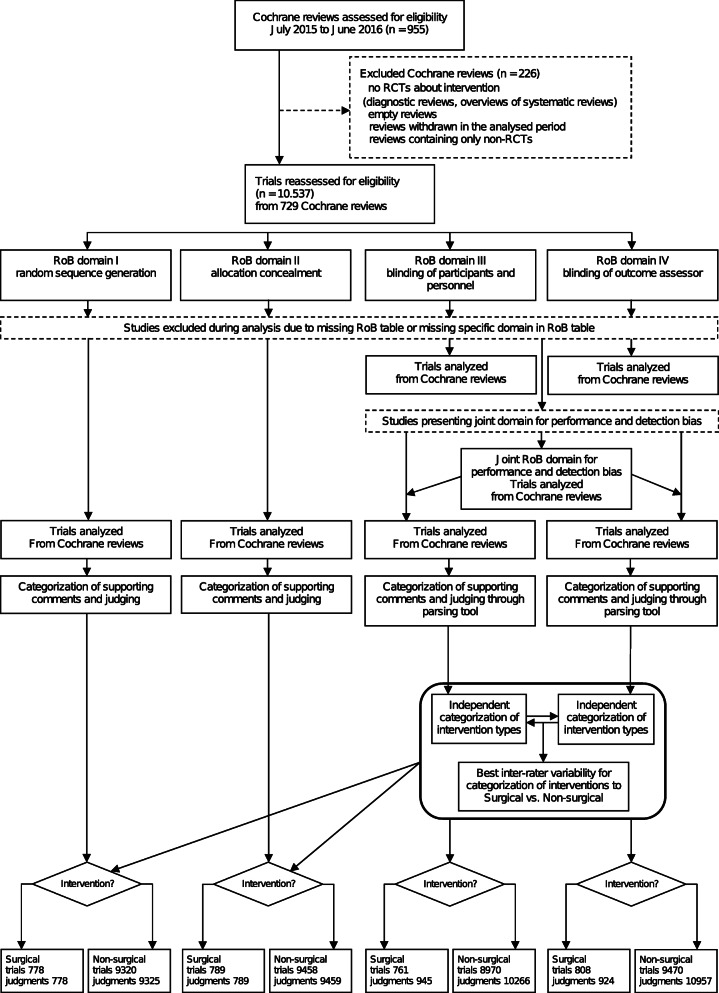
Flow diagram of the progress through the phases of the study and our previous studies

We analyzed 729 Cochrane reviews, with 10,537 included trials. The flow diagram is shown in Fig. 1. Not all reviews had analyzed all of the seven standard RoB domains; the random sequence generation domain was analyzed in trials from 709 reviews, allocation concealment domain from 717 reviews, blinding of participants and personnel domain from 685 reviews and blinding of outcome assessors domain from 721 reviews (Table 1, Fig. 1). In 171 analyzed reviews, Cochrane authors used the joint (single) domain for assessing blinding of participants, personnel, and outcome assessors.

**Table 1:** Number and proportion of Cochrane reviews included, trials missing data for specific domains, trials observed and judgments analyzed in total and according to types of intervention

		Domain I (random sequence generation)	Domain II (allocation concealment)	Domain III + ^a^ (blinding of participants and personnel)	Domain IV + ^a^ (blinding of outcome assessors)
**Reviews included (from original 729 reviews)**	**Surgical studies N (% of included)**	77 (10.9%)	79 (10.7%)	72 (10.5%)	79 (11.0%)
	**Non-surgical studies N (% of included)**	652 (92.0%)	659 (89.3%)	613 (89.5%)	642 (89.0%)
	**Total N (%)**	709 (97.3%)	717 (98.4%)	685 (94.0%)	721 (98.9%)
**Studies with domain missing (from original 10,537 studies)**	**Surgical N (%)**	37 (0.4%)	28 (0.3%)	54 (0.5%)	16 (0.2%)
	**Non-surgical N (%)**	402 (3.8%)	329 (3.1%)	752 (7.1%)	417 (4.0%)
	**Overall N (%)**	439 (4.2%)	357 (3.4%)	806 (7.6%)	433 (4.1%)
**Studies observed (from original 10,537 studies)**	**Surgical N (%)**	778 (7.4%)	789 (7.5%)	761 (7.2%)	808 (7.7%)
	**Non-surgical N (%)**	9320 (88.5%)	9391 (89.1%)	8970 (85.1%)	9296 (88.2%)
	**Overall N (%)**	10,098 (95.8%)	10,180 (96.6%)	9731 (92.4%)	10,104 (95.9%)
**Judgments analyzed (from number of observed studies in domain)**	**Surgical N (%)**	778 (7.7%)	789 (7.7%)	945 (8.4%)	924 (7.8%)
	**Non-surgical N (%)**	9325 (92.3%)	9459 (92.3%)	10,266 (91.6%)	10,957 (92.2%)
	**Surgical + N (+%)**^b^	0 (+ 0.0%)	0 (+ 0.0%)	184 (+ 19.5%)	116 (+ 12.6%)
	**Non-surgical + N (+%)**^b^	5 (+ 0.1%)	68 (+ 0.7%)	1296 (+ 12.6%)	1661 (+ 15.2%)
	**Overall N (+%)**^b^	10,103 (+ 0.0%)	10,248 (+ 0.7%)	11,211 (+ 13.2%)	11,881 (+ 15.0%)

^a^Also includes data for joint domain of blinding of participants, personnel, and outcome assessor

^b^Cochrane authors provided additional multiple judgments (for different outcomes)

Domain for blinding of participants and personnel was present in significantly fewer reviews (*n* = 685) compared to the other three analyzed domains (*P <* 0.001, Table 1). There was no difference in the prevalence of the usage of four analyzed RoB domains between surgical and non-surgical reviews (ranging from 10.5 to 11.0% and 89.0 to 92.0% respectively).

The highest variability was detected in the proportion of absent analyzed domains in trials of non-surgical interventions (*P* < 0.001, Table 1). This was best observed in the blinding of participants and personnel domain with more than 7% of cases missing this domain.

The number of judgments was higher in both domains about blinding compared to domains regarding randomization and allocation concealment due to Cochrane authors providing multiple judgments (for different outcomes). Thus, the overall number of analyzed judgments exceeded the total number of observed trials, but this was proportional in both surgical and non-surgical groups (*P* = 0.129, Table 1).

### Categorization of interventions

In the final categorization of interventions by two independent raters, inter-rater agreement for categorization of trials to surgical and non-surgical was almost perfect (Cohen’s Kappa 0.83, 95% CI [0.81 to 0.85]); details in Supplementary Table 1.

### Distribution and adequacy of judgments

The distribution of the different risk of bias categories (high/low/unclear) assigned by the Cochrane authors (see Table 2) for surgical vs. non-surgical trials did not differ for the randomization and allocation concealment domains (*P* = 0.409, *P* = 0.964, respectively), but differed significantly in the two domains about blinding (*P* < 0.001, Supplementary Table 2).

**Table 2:** Distribution of judgments by Cochrane authors and judgments reassessed in our studies by Cochrane Handbook according to the intervention (surgical/non-surgical)

RoB domain	Re-assessment of judgments in surgical trials					Re-assessment of judgments in non-surgical trials				
**I - random sequence generation**	**High** **risk**	**Low** **risk**	**Unclear** **risk**	**Total**	**Adequacy** **(95% CI)**	**High** **risk**	**Low** **risk**	**Unclear** **risk**	**Total**	**Adequacy** **(95% CI)**
Judgment by Cochrane authors										
High risk	31		1	32 (4.1%)	96.9%	233	20	80	333 (3.6%)	70.0%
Low risk		337	58	395 (50.8%)	85.3%	9	3631	916	4556 (48.9%)	79.7%
Unclear risk	4	17	330	351 (45.1%)	94.0%	28	87	4321	4436 (47.6%)	97.4%
**Total N (%)**	**35 (4.5%)**	**354 (45.5%)**	**389 (50.0%)**	**778 (100.0%)**	**89.7% (87.4, 91.8)**	**270 (2.9%)**	**3738 (40.1%)**	**5317 (57.0%)**	**9325 (100.0%)**	**87.8% (87.1, 88.4)**
**II - allocation concealment**	**High** **risk**	**Low** **risk**	**Unclear** **risk**	**Total**	**Adequacy** **(95% CI)**	**High** **risk**	**Low** **risk**	**Unclear** **risk**	**Total**	**Adequacy** **(95% CI)**
Judgment by Cochrane authors										
High risk	34		9	43 (5.4%)	79.1%	353		159	512 (5.4%)	68.9%
Low risk		83	187	270 (34.2%)	30.7%	17	746	2479	3242 (34.3%)	23.0%
Unclear risk			476	476 (60.3%)	100.0%	16	10	5679	5705 (60.3%)	99.5%
**Total N (%)**	**34 (4.3%)**	**83 (10.5%)**	**672 (85.2%)**	**789 (100.0%)**	**75.2% (72.0, 78.1)**	**386 (4.1%)**	**756 (8.0%)**	**8317 (87.9%)**	**9459 (100.0%)**	**71.7% (70.7, 72.6)**
**III +** ^a^ **- blinding of participants and personnel**	**High** **risk**	**Low** **risk**	**Unclear** **risk**	**Total**	**Adequacy** **(95% CI)**	**High** **risk**	**Low** **risk**	**Unclear** **risk**	**Total**	**Adequacy** **(95% CI)**
Judgment by Cochrane authors										
High risk	266	1	40	307 (32.5%)	86.6%	3392	17	350	3759 (36.6%)	90.2%
Low risk	25	108	59	192 (20.3%)	56.3%	130	1578	2006	3714 (36.2%)	42.5%
Unclear risk	49	20	377	446 (47.2%)	84.5%	505	87	2201	2793 (27.2%)	78.8%
**Total N (%)**	**340 (36.0%)**	**129 (13.7%)**	**476 (50.4%)**	**945 (100.0%)**	**79.5% (76.8, 82.0)**	**4027 (39.2%)**	**1682 (16.4%)**	**4557 (44.4%)**	**10,266 (100.0%)**	**69.9% (69.0, 70.7)**
**IV +** ^a^ **- blinding of outcome assessor**	**High** **risk**	**Low** **risk**	**Unclear** **risk**	**Total**	**Adequacy** **(95% CI)**	**High** **risk**	**Low** **risk**	**Unclear** **risk**	**Total**	**Adequacy** **(95% CI)**
Judgment by Cochrane authors										
High risk	174	1	44	219 (23.7%)	79.5%	2003	34	359	2396 (21.9%)	83.6%
Low risk	7	115	70	192 (20.8%)	59.9%	88	2374	2002	4464 (40.7%)	53.2%
Unclear risk	33	20	460	513 (55.5%)	89.7%	356	212	3529	4097 (37.4%)	86.1%
**Total N (%)**	**214 (23.2%)**	**136 (14.7%)**	**574 (62.1%)**	**924 (100.0%)**	**81.1% (78.4, 83.5)**	**2447 (22.3%)**	**2620 (23.9%)**	**5890 (53.8%)**	**10,957 (100.0%)**	**72.2% (71.3, 73.0)**

^a^Also includes data for joint domain of blinding of participants, personnel, and outcome assessor

The distribution of RoB judgments that we have made de novo*,* based on explanatory comments from RoB tables, was significantly different between surgical and non-surgical trials in the domain for randomization and the domain for blinding of outcome assessors (*P* = 0.022, *P* < 0.001 respectively, Supplementary Table 2). It almost reached the level of statistical difference for domain regarding allocation concealment (*P* = 0.069).

The prevalence of adequate judgments significantly varied between the four RoB domains (Kruskal-Wallis test, *P* < 0.001, Supplementary Table 3). In the entire sample of analyzed reviews, the highest prevalence of adequate judgments by Cochrane authors was found in RoB domain for randomization (87.9, 95% CI [87.3 to 88.6%]), followed by the domain for blinding of outcome assessors (72.9, 95% CI [72.0 to 73.7%]), allocation concealment (71.9, 95% CI [71.0 to 72.8%]), and blinding of participants and personnel (70.7, 95% CI [69.8 to 71.5%]).

The prevalence of adequate RoB judgments for all analyzed RoB domains was generally higher in surgical trials than in non-surgical trials. For two RoB domains assessing blinding, this difference between surgical and non-surgical trials was statistically significant (*P* < 0.001), for allocation concealment test power, was too low (*P* = 0.039, beta < 0.8), while the difference between two types of trials was not significant for RoB domain regarding randomization (*P* = 0.124) (Supplementary Table 2).

### Basis for RoB judgment justification

Various comments were used to support RoB judgments in Cochrane reviews. In the RoB domain for randomization, we demonstrated the significantly different distribution of categories of different supporting comments in the surgical vs. non-surgical group (*P <* 0.001, Table 3, Domain I). For surgical trials, computerized randomization and inappropriate randomization were mentioned more frequent and failure to describe the randomization method was less frequent compared to non-surgical trials (Table 3, Supplementary Table 4).

**Table 3:** Differentiation of justifications (causes) for risk judgments with tests and interpretations

	Risk judgment justification basis	Intervention	
**Domain I** (random sequence generation)	**Categorization of supporting comments**	**Non-surgical**	**Surgical**
	Randomization not described	5317 (57.0%)	389 (50.0%)
	Random number table	815 (8.7%)	68 (8.7%)
	Computerized randomization	2597 (27.8%)	253 (32.5%)
	Mechanical randomization	326 (3.5%)	33 (4.2%)
	Inappropriate randomization	270 (2.9%)	35 (4.5%)
**Domain II** (allocation concealment)	**Type of allocation concealment**	**Non-surgical**	**Surgical**
	Central allocation	506 (5.3%)	32 (4.1%)
	Incomplete SNOSE	1117 (11.8%)	147 (18.6%)
	Not described/unclear	7200 (76.1%)	525 (66.5%)
	Open / predictable allocation	386 (4.1%)	34 (4.3%)
	SNOSE	250 (2.6%)	51 (6.5%)
**Domain III +** ^a^ (blinding of participants and personnel)	**Blinding achieved**	**Non-surgical**	**Surgical**
	Achieved	1239 (12.1%)	38 (4.0%)
	Not done or not possible	4318 (42.1%)	414 (43.8%)
	Probably done	4709 (45.9%)	493 (52.2%)
	**Outcome influenced by lack of blinding**	**Non-surgical**	**Surgical**
	Influenced	472 (4.6%)	21 (2.2%)
	Unknown	9340 (91.0%)	832 (88.0%)
	Not influenced	454 (4.4%)	92 (9.7%)
**Domain IV +** ^a^ (blinding of outcome assessors)	**Blinding achieved**	**Non-surgical**	**Surgical**
	Achieved	1722 (15.7%)	84 (9.1%)
	Probably done	6695 (61.1%)	609 (65.9%)
	Not done or not possible	2540 (23.2%)	231 (25.0%)
	**Outcome influenced by lack of blinding**	**Non-surgical**	**Surgical**
	Influenced	919 (8.4%)	31 (3.4%)
	Unknown	9045 (82.5%)	839 (90.8%)
	Not influenced	993 (9.1%)	54 (5.8%)

^a^Also includes data for joint domain of blinding of participants, personnel and outcome assessor; SNOSE = sequentially numbered sealed opaque envelopes

In the RoB domain for allocation concealment, we found a similar distribution of types of allocation concealment between surgical and non-surgical trials (Table 3, Domain II). However, in surgical trials (vs. non-surgical) we detected a larger proportion of comments stating allocation concealment was properly achieved with the use of “sequentially numbered opaque sealed envelopes” (SNOSE) and a lower proportion of unclearly described methods of allocation concealment.

Both RoB domains about blinding had a significantly different distribution of comments about whether the blinding of key individuals was achieved between surgical and non-surgical trials (*P* < 0.001, Table 3, Domain III and IV). Successful blinding was significantly less frequent in surgical vs. non-surgical trials, for participants and personnel (4.0% vs. 12.1%, *P* < 0.001), and for outcome assessors (9.1% vs. 15.7%, *P* < 0.001).

Some outcomes are more susceptible to bias due to lack of blinding compared to others; however, the susceptibility of outcomes to be influenced by lack of blinding was sometimes described in less than 10% of comments for both domains about blinding (Table 3). We analyzed whether there was a difference in the distribution of comments in which Cochrane authors included information if blinding of key individuals influenced an outcome. This distribution was significantly different between surgical and non-surgical trials only in the RoB domain about blinding of participants and personnel (*P <* 0.001, Table 3, Supplementary Table 4).

## Discussion

The main finding of our study is that RoB judgments for randomization, allocation concealment, and domains on blinding were more accurate in Cochrane reviews that assessed surgical trials, compared to reviews of non-surgical trials. Even though seven domains are obligatory parts of the 2011 Cochrane RoB tool, some of the four analyzed domains were frequently absent in analyzed reviews. The absence of the analyzed four domains was more frequent in reviews of non-surgical trials.

We have chosen to analyze only Cochrane reviews for two reasons. First, Cochrane reviews must follow Cochrane methods and the usage of the Cochrane RoB tool is mandatory for them. Second, we have shown previously that the majority of authors of non-Cochrane reviews used RoB assessment, and the majority of those used the Cochrane RoB tool; however, most of them used it inadequately [20]. Among 269 analyzed non-Cochrane reviews that used the Cochrane RoB tool, only 16 (5.9%) reported RoB results fully, i.e. reported both judgment and accompanying comment that supports the judgment [20]. Due to inadequate reporting, i.e. failure of the majority of non-Cochrane reviews to report both judgment and an explanatory comment, analysis of the adequacy of RoB assessment is hindered in non-Cochrane reviews. Nevertheless, our findings are relevant for both Cochrane and non-Cochrane reviews, precisely because most non-Cochrane systematic reviews use the Cochrane RoB tool.

The absence of some RoB domains in analyzed Cochrane reviews indicates that Cochrane authors decided to “customize” the Cochrane RoB tool by removing some of the domains from the default settings of the RoB table. This was not the only customization that we have observed. We also found that many Cochrane authors introduced sub-domains, i.e. multiple judgments for a single domain, based on different outcomes. In these cases, for a single RoB domain, Cochrane authors provided multiple judgments based on the types of outcomes, for example – one judgment for objective outcomes and one judgment for subjective outcomes. Another example of customization is when authors split the domain ‘blinding of participants and personnel’ into two domains – one for blinding of participants, and one for blinding of personnel. The rationale for this customization is different outcomes, and different key individuals involved in a trial, may yield different RoB assessments.

Thus, we found a higher number of judgments compared to the number of analyzed RoB domains, i.e. number of individual trials analyzed with these RoB domains. A particularly higher number of judgments was found in both RoB domains for blinding, which indicates that Cochrane authors wanted to emphasize the potential difference in the impact of the success of blinding on different types of outcomes.

We found that the RoB domain regarding blinding of participants and personnel had the lowest prevalence of adequate assessments. Furthermore, we found that both domains about blinding have a higher prevalence of adequate judgments in surgical trials. For the domain regarding blinding of participants and personnel, this could be because blinding of those individuals is difficult to achieve in surgical trials [21], which leads to more transparency in descriptions of methodology in surgical trials. Therefore, automatically, in surgical trials, there are less judgments of “low risk” of bias, which were associated with the lowest prevalence of adequate assessments.

Results from our previous studies on RoB judgments in systematic reviews indicated that it would be beneficial to split domain “blinding of participants and personnel” into two domains, one for participants, and one for personnel [7]. This was implemented in the RoB 2 tool [22], which is not yet implemented in all Cochrane protocols and reviews. We also found that this same domain would not benefit from the further splitting of the domain based on different outcomes [7].

On the contrary, for the domain regarding blinding of outcome assessors, we found that it would be beneficial to introduce sub-domains for objective versus subjective outcomes. This approach would decrease the number of undefined outcomes with a subsequent increase in the prevalence of adequate assessments [16]. We also found that length of comment impacts proper justification of an RoB judgment and its adequacy [7].

Our findings have two aspects: recommendations for conducting trials with surgical interventions (to reduce risk of bias) and practical solutions for RoB assessment tools (to ensure adequate RoB judgments of trials).

Even though this study did not aim at analyzing the methodological flaws of the surgical trials, there are some simple recommendations that can be generalized. The allocation sequence should always be randomized. Computer randomization is recommended as it has multiple benefits. If blocked randomization is considered, blocks should be larger (avoid blocks of four), usage of minimization is advisable for multiple strata, and each subgroup should be randomized separately. Another benefit of computerized randomization is its wide availability. Third-party centralized randomization also adds up to allocation concealment being secured until the end of the study. Thus, the usage of sequentially numbered opaque sealed envelopes should be rendered obsolete, especially since we demonstrated it is widely used or described incompletely [10]. If systematic review authors do not find information about in research reports regarding specific methods for randomization and allocation concealment, they should be careful to avoid making erroneous RoB judgment.

If it is not possible to blind key individuals, as it is the case in many trials of surgical interventions, steps should be taken to reduce the risk of bias at different levels. At the participant level, if sham surgery is unethical or not approved, and restricting information about the procedure to the patient is not possible, some simple measures should be used when planning the study as well as a detailed description supporting the RoB judgment [6]. These measures might include not mixing the groups of patients, concealing incisions with larger dressings, and providing a defined standard of care identical for both intervention and control groups. The last two help to reduce RoB when blinding of the surgeon is not achievable. However, an expertise-based setup might be used with multiple surgeons/teams performing the same procedure for the same group. All of this can be added to RoB analyzing software to reduce the final RoB judgment for these domains.

For outcome assessment, the availability of a blinded secondary team of surgeons or surgical nurses is crucial for achieving low RoB. However, in unblinded assessment, the susceptibility of an outcome to lack of blinding is the most important factor for the final RoB judgment. Therefore, all outcomes should be defined (description of a positive and negative event/criteria) prior to the commencement of the study. When a defined outcome is not objective an outcome assessor should be predetermined and the method of measuring the outcome addressed before the observations are recorded [21]. If none of the above is possible a duplicate assessment if advisable or at least a statement acknowledging the limitations. Potentially, introducing a drop-down menu with various types of outcomes in software for conducting systematic reviews, could assist with a better assessment of RoB in trials with different groups of outcomes.

Practical solutions that this study can offer to improve RoB judgments in Cochrane systematic reviews include the suggestion that customization of RoB table should not be allowed in the RevMan software used to produce Cochrane systematic reviews. Otherwise, the authors will continue to have an option to delete certain RoB domains that they perhaps consider irrelevant. Reporting the RoB tool completely, which implies the use of all RoB domains, and both judgment and comment for each domain is important for adequate assessment of trial methodology. Furthermore, our findings regarding the length of comments indicate that authors should be encouraged, and warned by the software, to provide more detailed descriptions of their judgments in the comment field of the RoB table. Interventions for enhancing editors’ and peer reviewers’ assessment of RoB judgments, as well as interventions for improving review authors’ appraisal of RoB, would be welcome. RoB assessments are used to provide review conclusions and in the GRADE approach for rating the certainty of evidence in systematic reviews. Thus, inadequate RoB judgments may translate into inadequate review conclusions and inadequate assessment of evidence certainty, resulting in erroneous recommendations for further research and practice.

A limitation of our study is that we have perhaps made inadvertent mistakes when assessing the adequacy of Cochrane authors’ judgments through available supporting comments. To reduce bias, we made independent assessments by two authors for each analyzed domain and sub-domain. Additionally, we included in the analysis only the first four domains of the Cochrane RoB tool, because instructions from the Cochrane Handbook for these four domains are better characterized compared to the remaining three domains [11–13].

Furthermore, the primary aim of the study was to evaluate differences in the number and adequacy of RoB judgments in studies with surgical intervention. Thus, the categorization of “surgical vs. non-surgical” interventions was chosen according to the Kappa statistic as a measure of inter-rater agreement. Although we did not focus on the actual level of agreement, we used it as a measure for a better definition of the groups to be compared. However, we must point out that the main drawback of this method is the fact that it can result in what is termed the ‘base rate problem’ and is sensitive to ‘true prevalence’ in the data. If the true prevalence of a population is high or low, agreement expected by chance increases, and the magnitude of Kappa goes down. Moreover, within the broad category of non-surgical interventions, there are many interventions (e.g., psychosocial interventions, psychotherapies, screening, etc.) that cannot be blinded, sharing the same problems for RoB assessment with surgical interventions. Further exploration of difficulties associated with RoB assessment in other interventions that may be difficult to blind is thus welcome.

## Conclusion

RoB judgments were more in line with instructions from the Cochrane Handbook when Cochrane reviews assessed surgical trials, compared to those that analyzed non-surgical interventions. However, many RoB judgments in Cochrane reviews of both surgical and non-surgical trials were not in line with the Cochrane Handbook; therefore, further steps are warranted to scrutinize RoB assessment in trials of both surgical and non-surgical interventions.

## Supplementary information

**Additional file 1.** STROBE Statement - Checklist of items that should be included in reports of cross-sectional studies

**Additional file 2**: **Table S1.** Inter rater raw agreement and variability for different categorizations od interventions

**Additional file 3**: **Table S2.** Overview of the hypotheses, outcome measures, statistical tests used and results.

**Additional file 4**: **Table S3.** Overview of the variability of the prevalence of adequate RoB judgments throughout RoB domains and according to the type of intervention in observed trials with statistical tests and pairwise comparisons

**Additional file 5**: **Table S4.** More detailed differentiation of justifications (causes) for risk judgments with tests and interpretations

## Data Availability

All data collected and analyzed within this study are available from the corresponding author on reasonable request.

## Abbreviations

CDSR: Cochrane Database of Systematic Reviews
CPAP: Continuous positive airway pressure
ECV: External cephalic version
ERCP: Endoscopic, retrograde cholangiopancreatography
EMS: Extra-mucosal resection
HFJV: High-frequency jet ventilation
RCT: Randomized controlled trial
RoB: Risk of bias
SNOSE: Sequentially numbered opaque sealed envelopes
