# Supplementary material for: Adequacy of risk of bias assessment in surgical vs non-surgical trials in Cochrane reviews: a methodological study
Source: BMC Med Res Methodol. 2020 Sep 29;20:240. doi: 10.1186/s12874-020-01123-7 (PMC7526117; doi:10.1186/s12874-020-01123-7)
Supplement: Supplementary file 1 — Additional file 1. STROBE Statement - Checklist of items that should be included in reports of cross-sectional studies [file 12874_2020_1123_MOESM1_ESM.docx]

**Supplementary file 1: STROBE Statement - Checklist of items that should be included in reports of *cross-sectional studies***

|  | Item No | Recommendation | Adherence to the checklist and position in manuscript |
| --- | --- | --- | --- |
| **Title and abstract** | 1 | (*a*) Indicate the study’s design with a commonly used term in the title or the abstract | P1-2, Title and Abstract |
|  |  | (*b*) Provide in the abstract an informative and balanced summary of what was done and what was found | P4-5, Abstract |
| Introduction | | |  |
| Background/rationale | 2 | Explain the scientific background and rationale for the investigation being reported | P6, Background section |
| Objectives | 3 | State specific objectives, including any prespecified hypotheses | P6, Background section; Paragraph 3-4 |
| Methods | | |  |
| Study design | 4 | Present key elements of study design early in the paper | P7, Methods section, subtitle: *Study design and protocol* |
| Setting | 5 | Describe the setting, locations, and relevant dates, including periods of recruitment, exposure, follow-up, and data collection | P7, Methods section, subtitles: *Inclusion and exclusion criteria, Screening for study eligibility* |
| Participants | 6 | (*a*) Give the eligibility criteria, and the sources and methods of selection of participants | P7, Methods section, subtitles: *Inclusion and exclusion criteria, Screening for study eligibility*  P9, Methods section, subtitle: *Data extraction* |
| Variables | 7 | Clearly define all outcomes, exposures, predictors, potential confounders, and effect modifiers. Give diagnostic criteria, if applicable | P7-8, Methods section, subtitle: *Definition and categorization of interventions*  P9-10, Methods section, subtitles: *Primary outcome, Secondary outcomes* |
| Data sources/ measurement | 8* | For each variable of interest, give sources of data and details of methods of assessment (measurement). Describe comparability of assessment methods if there is more than one group | P9-10, Methods section, subtitles: *Data extraction, Assessment of adequacy for four domains of risk of bias tool* |
| Bias | 9 | Describe any efforts to address potential sources of bias | P7, Methods section, subtitles: *Inclusion and exclusion criteria, Screening for study eligibility* |
| Study size | 10 | Explain how the study size was arrived at | Convenient sample based on our previous studies |
| Quantitative variables | 11 | Explain how quantitative variables were handled in the analyses. If applicable, describe which groupings were chosen and why | P7-10, Methods section, subtitles: *Definition and categorization of interventions, Primary outcome, Secondary outcomes, Data extraction, Assessment of adequacy for four domains of risk of bias tool* |
| Statistical methods | 12 | (*a*) Describe all statistical methods, including those used to control for confounding | P10, Methods section, subtitle: *Statistics*  Supplementary table 1: Inter rater raw agreement and variability for different categorizations od interventions  Supplementary table 2: Overview of the hypotheses, outcome measures, statistical tests used and results with DF and type I and II errors stated for parametric tests. |
|  |  | (*b*) Describe any methods used to examine subgroups and interactions | P10, Methods section, subtitle: *Statistics* |
|  |  | (*c*) Explain how missing data were addressed | P7-8, Methods section, subtitle: *Definition and categorization of interventions*  Flow diagram of the progress through the phases of the study and our previous studies  For all the data observed from our previous studies we did not have a missing variable. |
|  |  | (*d*) If applicable, describe analytical methods taking account of sampling strategy | Not applicable, convenient sample of Cochrane reviews based on our previous studies |
|  |  | (*e*) Describe any sensitivity analyses | Supplementary table 1: Inter rater raw agreement and variability for different categorizations od interventions  Supplementary table 2: Overview of the hypotheses, outcome measures, statistical tests used and results with DF and type I and II errors stated for parametric tests. |
| Results | | |  |
| Participants | 13* | (a) Report numbers of individuals at each stage of study—eg numbers potentially eligible, examined for eligibility, confirmed eligible, included in the study, completing follow-up, and analysed | P11 Results section |
|  |  | (b) Give reasons for non-participation at each stage | Not applicable |
|  |  | (c) Consider use of a flow diagram | Flow diagram of the progress through the phases of the study and our previous studies stated |
| Descriptive data | 14* | (a) Give characteristics of study participants (eg demographic, clinical, social) and information on exposures and potential confounders | P11 Results section  P12 Results section, subtitle: *Categorization of interventions* |
|  |  | (b) Indicate number of participants with missing data for each variable of interest | Not applicable |
| Outcome data | 15* | Report numbers of outcome events or summary measures |  |
| Main results | 16 | (*a*) Give unadjusted estimates and, if applicable, confounder-adjusted estimates and their precision (eg, 95% confidence interval). Make clear which confounders were adjusted for and why they were included | P12-14 Results section, subtitles: *Categorization of interventions, Distribution and adequacy of judgments, Basis for RoB judgment justification*  Results presented with accompanying 95%CI both in the tables and in the manuscript main text. |
|  |  | (*b*) Report category boundaries when continuous variables were categorized | P12, P14 Results section, subtitles: *Categorization of interventions, Basis for RoB judgment justification* |
|  |  | (*c*) If relevant, consider translating estimates of relative risk into absolute risk for a meaningful time period | Not relevant for the observed data |
| Other analyses | 17 | Report other analyses done—eg analyses of subgroups and interactions, and sensitivity analyses | Supplementary table 1 and 2 |
| Discussion | | |  |
| Key results | 18 | Summarise key results with reference to study objectives | P15-17 Discussion section, paragraphs 1-6 |
| Limitations | 19 | Discuss limitations of the study, taking into account sources of potential bias or imprecision. Discuss both direction and magnitude of any potential bias | P19 Discussion section, paragraphs 12-13 |
| Interpretation | 20 | Give a cautious overall interpretation of results considering objectives, limitations, multiplicity of analyses, results from similar studies, and other relevant evidence | P17-19 Discussion section, paragraphs 7-10 |
| Generalisability | 21 | Discuss the generalisability (external validity) of the study results | P17-19 Discussion section, paragraph 11 |
| Other information | | |  |
| Funding | 22 | Give the source of funding and the role of the funders for the present study and, if applicable, for the original study on which the present article is based | P21 Declarations section |

*Give information separately for exposed and unexposed groups.

**Note:** An Explanation and Elaboration article discusses each checklist item and gives methodological background and published examples of transparent reporting. The STROBE checklist is best used in conjunction with this article (freely available on the Web sites of PLoS Medicine at http://www.plosmedicine.org/, Annals of Internal Medicine at http://www.annals.org/, and Epidemiology at http://www.epidem.com/). Information on the STROBE Initiative is available at www.strobe-statement.org.
