# Supplementary material for: Adequacy of risk of bias assessment in surgical vs non-surgical trials in Cochrane reviews: a methodological study
Source: BMC Med Res Methodol. 2020 Sep 29;20:240. doi: 10.1186/s12874-020-01123-7 (PMC7526117; doi:10.1186/s12874-020-01123-7)
Supplement: Supplementary file 2 — Additional file 2: Table S1. Inter rater raw agreement and variability for different categorizations od interventions [file 12874_2020_1123_MOESM2_ESM.docx]

**Supplementary table 1: Inter rater raw agreement and variability for different categorizations of interventions**

| **Domain** | **Raw agreement** | **Cohen's Kappa** | **95%CI** | **Cohen's Kappa interpretation** |
| --- | --- | --- | --- | --- |
| Categories |  |  |  |  |
| **Domain III+*** (blinding of participants and personnel) |  |  |  |  |
| SURG vs MIXED vs CONS | 83.0% | 0.5744 | 0.5570 to 0.5918 | moderate agreement |
| SURG vs NON-SURG | 96.8% | 0.7691 | 0.7457 to 0.7925 | substantial agreement |
| CONS vs NON-CONS | 85.5% | 0.6117 | 0.5942 to 0.6292 | substantial agreement |
| MIXED vs NON-MIXED | 83.7% | 0.4305 | 0.4066 to 0.4544 | moderate agreement |
| **Domain IV+*** (blinding of outcome assessors) |  |  |  |  |
| SURG vs MIXED vs CONS | 87.1% | 0.6645 | 0.6488 to 0.6801 | substantial agreement |
| SURG vs NON-SURG | 97.8% | 0.8329 | 0.8128 to 0.8529 | almost perfect agreement |
| CONS vs NON-CONS | 88.9% | 0.6930 | 0.6774 to 0.7086 | substantial agreement |
| MIXED vs NON-MIXED | 87.5% | 0.5475 | 0.5261 to 0.5690 | moderate agreement |

* standard domain merged with joint domain for performance and detection bias; SURG = surgical intervention; MIXED = mixed or unclear intervention; CONS = conservative or non-invasive intervention; NON-SURG = all but surgical; NON-CONS = all but conservative; NON-MIXED = all but mixed
