# Supplementary material for: Adequacy of risk of bias assessment in surgical vs non-surgical trials in Cochrane reviews: a methodological study
Source: BMC Med Res Methodol. 2020 Sep 29;20:240. doi: 10.1186/s12874-020-01123-7 (PMC7526117; doi:10.1186/s12874-020-01123-7)
Supplement: Supplementary file 3 — Additional file 3: Table S2. Overview of the hypotheses, outcome measures, statistical tests used and results. [file 12874_2020_1123_MOESM3_ESM.docx]

## Supplementary table 2: Overview of the hypotheses, outcome measures, statistical tests used and results

| **Variable/Outcome** | **Null-hypothesis** | **Method of analysis** | **Result** | **Conclusion** |
| --- | --- | --- | --- | --- |
|  |  |  |  |  |
| *Main analysis: Overall proportion of adequate RoB judgments assigned by Cochrane authors* | Proportion of adequate RoB judgments is the same in groups of trials with surgical (89.7%) vs. non-surgical (87.8%) intervention in the domain for random sequence generation | Chi-squared test | *P* = 0.124  DF = 1 | There is not enough evidence to reject null hypothesis: no significant difference between groups. |
|  | Proportion of adequate RoB judgments is the same in groups of trials with surgical (75.2%) vs. non-surgical (71.7%) intervention in the domain for allocation concealment | Chi-squared test | *P* = 0.039  DF = 1  β < 0.8 | Test inconclusive: it seems there is are more adequate RoB judgments in surgical vs. non-surgical group, however power of the test is lower than 80%. |
|  | Proportion of adequate RoB judgments is the same in groups of trials with surgical (79.5%) vs. non-surgical (69.9%) intervention in the domain for blinding participants and personnel and joint blinding domain | Chi-squared test | *P* < 0.001  DF = 1 | Null hypothesis rejected: There are significantly more adequate RoB judgments in surgical vs. non- surgical group. |
|  | Proportion of adequate RoB judgments is the same in groups of trials with surgical (81.1%) vs. non-surgical (72.2%) intervention in the domain for blinding of outcome assessors and joint blinding domain | Chi-squared test | *P* < 0.001  DF = 1 | Null hypothesis rejected: There are significantly more adequate RoB judgments in surgical vs. non- surgical group. |
| *Secondary analysis:* *Number and proportion of Cochrane reviews* | Distribution of surgical vs. non-surgical reviews within domain is the same between all four RoB domains | Kruskal-Wallis test | *P* < 0.001  DF = 3 | Null hypothesis rejected: significant difference between groups. Post-hoc Conover test detected no pairwise differences so one-way ANOVA was reapplied. |
|  | Distribution of surgical vs. non-surgical reviews within domain is the same between all four RoB domains. | One-way ANOVA | *P* < 0.001  DF = 3 | Null hypothesis rejected: significant difference between groups. Post-hoc Student-Newman-Keuls test detected domain for blinding of participants and personnel showed significantly different distribution of surgical vs. non-surgical reviews. This domain was present in significantly less reviews than other domains. |
|  | Prevalence of the usage of four analyzed RoB domains is the same between surgical and non-surgical reviews. | Kruskal-Wallis | *P* = 0.843  DF = 1 | There is not enough evidence to reject null hypothesis: no significant difference between groups. |
| *Secondary analysis:* *Number and proportion of trials missing data for specific domains and trials observed according to types of intervention* | Distribution of surgical vs. non-surgical trials with domains missing or observed is the same between all four RoB domains. | Kruskal-Wallis | *P* < 0.001  DF = 3 | Null hypothesis rejected: significant difference between groups. Post-hoc Conover test detected domain for blinding of participants and personnel showed significantly different distribution of surgical vs. non-surgical reviews. This domain was present in significantly less trials than other domains. |
|  | Prevalence of the usage of four analyzed RoB domains is the same between surgical and non-surgical trials. | Kruskal-Wallis | *P* < 0.001  DF = 3 | Null hypothesis rejected: significant difference between groups. Post-hoc Conover test detected group of non-surgical trials missing the domain to be different from all other groups. This group demonstrated most variability. |
| *Secondary analysis:* *Number and proportion of judgments analyzed according to types of intervention* | Distribution of judgments for surgical vs. non-surgical trials observed is the same between all four RoB domains. | Kruskal-Wallis | *P* = 0.129  DF = 3 | There is not enough evidence to reject null hypothesis: no significant difference between groups. |
| *Secondary analysis:* *Distribution of risk judgments (high/low/unclear) assigned by Cochrane authors* | Distribution is the same in group of trials with surgical vs. non-surgical intervention in the domain for random sequence generation | Mann-Whitney | *P* = 0.409 | There is not enough evidence to reject null hypothesis: no significant difference between groups. |
|  | Distribution is the same in group of trials with surgical vs. non-surgical intervention in the domain for allocation concealment | Mann-Whitney | *P* = 0.964 | There is not enough evidence to reject null hypothesis: no significant difference between groups. |
|  | Distribution is the same in group of trials with surgical vs. non-surgical intervention in the domain for blinding participants and personnel and joint blinding domain | Mann-Whitney | *P* < 0.001 | Null hypothesis rejected: There is less low RoB judgments and more unclear risk judgments in surgical vs. non-surgical group. |
|  | Distribution is the same in group of trials with surgical vs. non-surgical intervention in the domain for blinding of outcome assessors and joint blinding domain | Mann-Whitney | *P* < 0.001 | Null hypothesis rejected: There is less low risk RoB judgments and more unclear risk RoB judgments in surgical vs. non-surgical group. |
| *Secondary analysis: Distribution of risk judgments (high/low/unclear) calculated in our studies* | Distribution is the same in group of trials with surgical vs. non-surgical intervention in the domain for random sequence generation | Mann-Whitney | *P* = 0.022 | Null hypothesis rejected: There is more low and high risk RoB judgments, due to less unclear risk RoB judgments in surgical vs. non-surgical group. |
|  | Distribution is the same in group of trials with surgical vs. non-surgical intervention in the domain for allocation concealment | Mann-Whitney | *P* = 0.069 | There is not enough evidence to reject null hypothesis: no significant difference between groups. |
|  | Distribution is the same in group of trials with surgical vs. non-surgical intervention in the domain for blinding participants and personnel and joint blinding domain | Mann-Whitney | *P* = 0.559 | There is not enough evidence to reject null hypothesis: no significant difference between groups. |
|  | Distribution is the same in group of trials with surgical vs. non-surgical intervention in the domain for blinding of outcome assessors and joint blinding domain | Mann-Whitney | *P* < 0.001 | Null hypothesis rejected: There is less low risk RoB judgments and more unclear risk RoB judgments in surgical vs. non-surgical group |
| *Secondary analysis: Successful blinding of key individuals* | Prevalence of successful blinding of participants and personnel is the same in the groups of trials with surgical intervention (4.0%) vs. non-surgical (12.1%) intervention. | Chi-squared test | *P* < 0.001  DF = 1 | Null hypothesis rejected: Successful blinding for participants and personnel is significantly less frequent in surgical vs. non-surgical trials. |
|  | Prevalence of successful blinding of outcome assessors is the same in the groups of trials with surgical intervention (9.1%) vs. non-surgical (15.7%) intervention. | Chi-squared test | *P* < 0.001  DF = 1 | Null hypothesis rejected: Successful blinding for outcome assessors is significantly less frequent in surgical vs. non-surgical trials. |

Acronyms: DF = degrees of freedom.
