# Supplementary material for: Adequacy of risk of bias assessment in surgical vs non-surgical trials in Cochrane reviews: a methodological study
Source: BMC Med Res Methodol. 2020 Sep 29;20:240. doi: 10.1186/s12874-020-01123-7 (PMC7526117; doi:10.1186/s12874-020-01123-7)
Supplement: Supplementary file 4 — Additional file 4: Table S3. Overview of the variability of the prevalence of adequate RoB judgments throughout RoB domains and according to the type of intervention in observed trials with statistical tests and pairwise comparisons [file 12874_2020_1123_MOESM4_ESM.docx]

## Supplementary table 3: Overview of the variability of the prevalence of adequate RoB judgments throughout RoB domains and according to the type of intervention in observed trials with statistical tests and pairwise comparisons

| **Domain - type of intervention in trials** | | **Adequacy** | **Definition test** | **Post hoc pairwise comparison test** |
| --- | --- | --- | --- | --- |
| **Factor number** | **Domain - surgical intervention** |  | **Kruskal-Wallis, P < 0.001** | **Conover, *P* < 0.05** |
| #1 | Randomization | 698/778 (89.7%) | significant difference in proportions of adequate RoB judgments between the domains | different from factor #2 #3 #4 |
| #2 | Allocation concealment | 593/789 (75.2%) |  | different from factor #1 #4 |
| #3 | Blinding of participants and personnel* | 751/945 (79.5%) |  | different from factor #1 |
| #4 | Blinding of outcome assessors* | 749/924 (81.1%) |  | different from factor #1 #2 |
| **Factor number** | **Domain - non-surgical intervention** |  | **Kruskal-Wallis, P < 0.001** | **Conover, *P* < 0.05** |
| #1 | Randomization | 8185/9325 (87.8%) | significant difference in proportions of adequate RoB judgments between the domains | different from factor #2 #3 #4 |
| #2 | Allocation concealment | 6778/9459 (71.7%) |  | different from factor #1 #3 |
| #3 | Blinding of participants and personnel* | 7171/10266 (69.9%) |  | different from factor #1 #2 #4 |
| #4 | Blinding of outcome assessors* | 7906/10957 (72.2%) |  | different from factor #1 #3 |
| **Factor number** | **Domains - overall** |  | **Kruskal-Wallis, P < 0.001** | **Conover, *P* < 0.05** |
| #1 | Randomization | 8883/10103 (87.9%) | significant difference in proportions of adequate RoB judgments between the domains | different from factor #2 #3 #4 |
| #2 | Allocation concealment | 7371/10248 (71.9%) |  | different from factor #1 |
| #3 | Blinding of participants and personnel* | 7922/11211 (70.7%) |  | different from factor #1 #4 |
| #4 | Blinding of outcome assessors* | 8655/11881 (72.8%) |  | different from factor #1 #3 |

*also includes data for joint domain of blinding of participants, personnel, and outcome assessor
