# Supplementary material for: Adequacy of risk of bias assessment in surgical vs non-surgical trials in Cochrane reviews: a methodological study
Source: BMC Med Res Methodol. 2020 Sep 29;20:240. doi: 10.1186/s12874-020-01123-7 (PMC7526117; doi:10.1186/s12874-020-01123-7)
Supplement: Supplementary file 5 — Additional file 5: Table S4. More detailed differentiation of justifications (causes) for risk judgments with tests and interpretations [file 12874_2020_1123_MOESM5_ESM.docx]

**Supplementary table 4: Differentiation of justifications (causes) for risk judgments with tests and interpretations.**

|  | **Risk judgment justification basis** | | **Intervention** | | **Discrimination test** | **Definition test** | **Post hoc pairwise comparison test** |
| --- | --- | --- | --- | --- | --- | --- | --- |
| **Domain I**  (random sequence generation) | **Factor number** | **Categorization of supporting comments** | **Non-surgical** | **Surgical** | **Mann-Whitney, *P* < 0.001** | **ANOVA, *P* = 0.001** | **Student-Newman-Keuls, *P* < 0.05** |
|  | #1 | Randomization not described | 5317 (57.0%) | 389 (50.0%) | difference in distribution of categories between groups | difference in proportions between categories | different from factor #3 #5 |
|  | #2 | Random number table | 815 (8.7%) | 68 (8.7%) |  |  |  |
|  | #3 | Computerized randomization | 2597 (27.8%) | 253 (32.5%) |  |  | different from factor #1 |
|  | #4 | Mechanical randomization | 326 (3.5%) | 33 (4.2%) |  |  |  |
|  | #5 | Inappropriate randomization | 270 (2.9%) | 35 (4.5%) |  |  | different from factor #1 |
| **Domain II** (allocation concealment) | **Factor number** | **Type of allocation concealment** | **Non-surgical** | **Surgical** | **Mann-Whitney, *P* = 0.482** | **Kruskal-Wallis, *P* < 0.001** | **Conover, *P* < 0.05** |
|  | #1 | Central allocation | 506 (5.3%) | 32 (4.1%) | no different distribution between groups | difference in proportions between types of allocation concealment | different from factor #5 |
|  | #2 | Incomplete SNOSE | 1117 (11.8%) | 147 (18.6%) |  |  | different from factor #3 |
|  | #3 | Not described/unclear | 7200 (76.1%) | 525 (66.5%) |  |  | different from factor #2 #5 |
|  | #4 | Open / predictable allocation | 386 (4.1%) | 34 (4.3%) |  |  | different from factor #5 |
|  | #5 | SNOSE | 250 (2.6%) | 51 (6.5%) |  |  | different from factor #1 #3 #4 |
| **Domain III+*** (blinding of participants and personnel) | **Factor number** | **Blinding achieved** | **Non-surgical** | **Surgical** | **Mann-Whitney, *P* < 0.001** | **Kruskal-Wallis, *P* < 0.001** | **Conover, *P* < 0.05** |
|  | #1 | Achieved | 1239 (12.1%) | 38 (4.0%) | different distribution between groups | difference in proportions of achievement | different from factor #2 #3 |
|  | #2 | Not done or not possible | 4318 (42.1%) | 414 (43.8%) |  |  | different from factor #1 |
|  | #3 | Probably done | 4709 (45.9%) | 493 (52.2%) |  |  | different from factor #1 |
|  | **Factor number** | **Outcome influenced by lack of blinding** | **Non-surgical** | **Surgical** | **Mann-Whitney, *P* < 0.001** | **Kruskal-Wallis, *P* < 0.001** | **Conover, *P* < 0.05** |
|  | #1 | Influenced | 472 (4.6%) | 21 (2.2%) | different distribution between groups | difference in proportions of influence | different from factor #3 |
|  | #2 | Unknown | 9340 (91.0%) | 832 (88.0%) |  |  | different from factor #3 |
|  | #3 | Not influenced | 454 (4.4%) | 92 (9.7%) |  |  | different from factor #1 #2 |
| **Domain IV+*** (blinding of outcome assessors) | **Factor number** | **Blinding achieved** | **Non-surgical** | **Surgical** | **Mann-Whitney, *P* < 0.001** | **Kruskal-Wallis, *P* < 0.001** | **Conover, *P* < 0.05** |
|  | #1 | Achieved | 1722 (15.7%) | 84 (9.1%) | different distribution between groups | difference in proportions of achievement | different from factor #2 #3 |
|  | #2 | Probably done | 6695 (61.1%) | 609 (65.9%) |  |  | different from factor #1 |
|  | #3 | Not done or not possible | 2540 (23.2%) | 231 (25.0%) |  |  | different from factor #1 |
|  | **Factor number** | **Outcome influenced by lack of blinding** | **Non-surgical** | **Surgical** | **Mann-Whitney, *P* = 0.206** | **Kruskal-Wallis, *P* < 0.001** | **Conover, *P* < 0.05** |
|  | #1 | Influenced | 919 (8.4%) | 31 (3.4%) | no different distribution between groups | difference in proportions of influence | different from factor #2 |
|  | #2 | Unknown | 9045 (82.5%) | 839 (90.8%) |  |  | different from factor #1 |
|  | #3 | Not influenced | 993 (9.1%) | 54 (5.8%) |  |  |  |

*also includes data for joint domain of blinding of participants, personnel and outcome assessor; SNOSE = sequentially numbered sealed opaque envelopes
